# DiffErential attainment and Factors AssoCiated with Training applications and Outcomes (DE FACTO) for general surgery applications in the UK: retrospective study

**DOI:** 10.1093/bjsopen/zrae166

**Published:** 2025-01-30

**Authors:** Sarika Grover, Siddarth Raj, Martina Spazzapan, Beth Russell, Harroop Bola, Noel Biju, Sachin Malde, Simon Fleming, Stella Vig

**Affiliations:** King’s College London School of Medicine, London, UK; University Hospitals Coventry and Warwickshire NHS Trust, Coventry, UK; King’s College London School of Medicine, London, UK; University Hospitals Coventry and Warwickshire NHS Trust, Coventry, UK; Department of Urology, Guy’s and St Thomas’ Hospitals NHS Trust, London, UK; King’s College London School of Cancer & Pharmaceutical Sciences, London, UK; Imperial College Faculty of Medicine, London, UK; King’s College London School of Medicine, London, UK; Department of Urology, Guy’s and St Thomas’ Hospitals NHS Trust, London, UK; Hand Surgery Unit, Royal North Shore Hospital, St Leonards, New South Wales, Australia; Department of Vascular and General Surgery, Croydon Health Services NHS Trust, London, UK

The process of securing a core surgical trainee (CST) position in the UK and Ireland remains highly competitive due to a limited number of posts and an increasing number of applicants. CSTs are at least 2 years postgraduate from their medical degree. In 2023, 2539 candidates competed for 609 CST posts in the UK, resulting in a competition ratio of 4.17, an increase compared with previous years^1^. Upon completing CST become eligible to apply for Higher Surgical Training (ST3); however, the number of ST3 posts, particularly in general surgery, has not kept up with increasing demand. For instance, in 2021, 607 candidates applied for 136 ST3 positions, a competition ratio of 4.46.

Despite recent interest in fostering diversity within surgical specialties, particularly in addressing sex disparities, there remains a paucity of data on the demographic, socioeconomic and educational factors influencing both application and success rates for ST3 positions. This study seeks to address these knowledge gaps by analysing demographic, socioeconomic and educational characteristics associated with CSTs who apply for and receive offers for ST3 in general surgery.

This retrospective study analysed data from the UK Medical Education Database (UKMED) between January 2014 and December 2019. Primary outcomes were applying to and receiving an offer for a general surgery ST3 post on the first attempt. Adjusted logistic regression models were used to calculate odds ratios (ORs) for variables including age, sex, ethnicity, socioeconomic status (SES), domicile status, medical school type, Situational Judgement Test (SJT) scores and postgraduate exam success. This methodology has been previously utilized for other specialties^2,3^. Further information can be found in the *Supplementary material*.

In this cohort of 1960 CSTs, the majority were male (63%), UK domiciled before medical school (81%) and attended Russell Group universities (69%). Of the 706 first-time applicants for general surgery ST3 positions, 477 (67.6%) received an offer. Female trainees were more likely to both apply and secure offers in general surgery (OR 1.82, 95% c.i. 1.51 to 2.20; OR 1.73, 95% c.i. 1.25 to 2.39), as were Russell Group graduates (OR 2.22, 95% c.i. 1.41 to 3.48) (*Table 1*). Higher SJT scores were also associated with offer success. Those who identified as black and minority ethnic (BME) were less likely to receive offers in comparison to their ‘white’ counterparts (OR 0.72, 95% c.i. 0.51 to 0.99; *P* = 0.044) (*Table 1*). Age and multiple specialty applications showed limited or no impact on application and offer rate (*Table 1*).

**Table 1:** Odds ratios and 95% confidence intervals (c.i.) for factors associated with applying to and obtaining a higher surgical training post in general surgery (*n* = 706)

Variable	Number of applicants	OR* (95% c.i.)	Number offered	OR* (95% c.i.)
**Decade of birth at time of application**				
1960s	0	1.00 (Ref.)	0	N/A (N/A)
1970s	10	0.89 (0.05,16.67)	5	1.00 (Ref.)
1980s	480	0.55 (0.03,8.89)	330	2.16 (0.53,8.77)
1990s	215	0.57 (0.04,9.18)	140	2.00 (0.49,8.23)
**Sex**				
Male	385	1.00 (Ref.)	240	1.00 (Ref.)
Female	325	1.82† (1.51,2.20)	240	1.73† (1.25,2.39)
**Ethnicity**				
White	385	1.00 (Ref.)	275	1.00 (Ref.)
BME	270	1.08 (0.89,1.32)	170	0.72† (0.51,0.99)
**IMD**				
1	30	1.00 (Ref.)	20	1.00 (Ref.)
2	70	1.98† (1.12,3.46)	40	0.83 (0.32,2.16)
3	90	1.09 (0.65,1.85)	70	1.33 (0.51,3.45)
4	125	1.19 (0.71,1.98)	95	1.36 (0.55,3.41)
5	200	1.15 (0.71,1.88)	140	1.20 (0.50,2.89)
**Domicile**				
Non UK	90	1.00 (Ref.)	375	1.00 (Ref.)
UK	525	0.60† (0.44,0.82)	60	1.46 (0.89,2.42)
**Graduate on entry to medical school**				
Graduate	100	1.00 (Ref.)	65	1.00 (Ref.)
Not a graduate	515	0.92 (0.60,1.43)	370	0.83 (0.38,1.83)
**POLAR quintile**				
1	15	1.00 (Ref.)	15	1.00 (Ref.)
2	40	1.00 (0.48,2.04)	25	0.66 (0.36,3.85)
3	100	1.39 (0.72,2.70)	65	0.61 (0.33,2.93)
4	120	0.99 (0.52,1.88)	85	0.88 (0.32,3.56)
5	240	0.96 (0.51,1.79)	180	N/A (N/A)
**Russell Group medical school**				
Russell Group	465	1.00 (Ref.)	95	1.00 (Ref.)
Non-Russell Group	150	1.03 (0.79,1.33)	340	2.22† (1.41,3.48)
**SJT score**				
<35	20	1.00 (Ref.)	25	1.00 (Ref.)
35–39	185	1.17 (0.64,2.16)	140	3.67† (1.98,12.50)
40–44	195	1.32 (0.72,2.44)	135	3.41† (1.02,11.47)
≥45	20	2.30 (0.98,5.43)	10	1.77 (0.58,13.40)
**SJT attempts**				
1	520	1.00 (Ref.)	N/A	1.00 (Ref.)
2	0	0.35 (0.04,2.95)	N/A	N/A (N/A)
**Multiple applications**				
Applied to 1 specialty	640	8.19‡ (3.47,11.70)	40	1.00 (Ref.)
Applied to >1 specialty	65	1.00 (Ref.)	0	0.33 (0.14,0.79)

BME, black and minority ethnic; IMD, Index of Multiple Deprivation; N/A, not applicable; POLAR, ‘Participation of local areas’ which is metric of young people entering higher education at age 18 or 19 years of age; SJT, Situational Judgement Test. *Denotes adjusted odds ratio(s) as defined by the DAG graph (*Supplementary Figure 1*). †Denotes statistical significance that is *P*  *<* 0.05. ‡Denotes cases where the presence of collinearity rendered precise estimation unattainable.

In the context of adversity within a conventionally male-dominated profession, these findings highlight an encouraging trend. Specifically, there is an observed increase in both the propensity of female applicants applying for general surgery and the likelihood of receiving offers. This suggests a positive shift toward greater sex inclusivity within the field. Observational data from 2011 to 2020 corroborate our data, revealing a temporal increase in female representation among both registrars and consultants in general surgery^4^. Based on these findings, sex parity within the profession is projected to be achieved by 2028^4^. Despite this, significant challenges persist for BME applicants. These include disproportionately lower pass rates in specialty examinations, an increased likelihood of receiving unsatisfactory annual review of competence progression outcomes, continued underrepresentation in surgical leadership roles and pervasive racial discrimination^5^. Evidence suggests that an SJT score between 35 and 44 was associated with improved odds. However, as of 2024, The United Kingdom Foundation Programme Office has replaced the SJT with a preference-informed allocation system, negating the impact of SJT performance as a differential predictor for application success.

Overall, this mixed picture highlights the successes of sex-related initiatives but provides evidence suggesting ethnic disparity in UK general surgical training. Further high-quality, prospective research is needed as we work towards developing a diverse surgical workforce.

## Supplementary Material

zrae166_Supplementary_Data

## Data Availability

Owing to restrictions on participant consent and data-sharing policies, the supporting data are not publicly available.
