# Supplementary material for: DiffErential attainment and Factors AssoCiated with Training applications and Outcomes (DE FACTO) for general surgery applications in the UK: retrospective study
Source: BJS Open. 2025 Jan 30;9(1):zrae166. doi: 10.1093/bjsopen/zrae166 (PMC11780877; doi:10.1093/bjsopen/zrae166)
Supplement: zrae166_Supplementary_Data [file zrae166_supplementary_data.docx]

**DiffErential attainment and Factors AssoCiated with Training applications and Outcomes (DE FACTO) for General Surgery applications in the United Kingdom: Retrospective Study**

Sarika Grover MBBS^1,2*^, Siddarth Raj MRCS^1,2*^, Martina Spazzapan MRCS^3^, Beth Russell PhD^4^, Harroop Bola BSc^5^, Noel Biju^1^, Sachin Malde FRCS^3^, Simon Fleming FRCS^6^, Stella Vig FRCS^7^

^1^King's College London School of Medicine, London, UK.

^2^University Hospitals Coventry and Warwickshire NHS Trust, Coventry, UK.

^3^ Guy's and St Thomas' Hospitals NHS Trust, Department of Urology, London, UK.

^4^King's College London School of Cancer & Pharmaceutical Sciences, London UK.

^5^Imperial College Faculty of Medicine, London, UK

^6^Hand Surgery Unit, Royal North Shore Hospital, St Leonards, New South Wales, Australia

^7^Department of Vascular and General Surgery, Croydon Health Services NHS Trust, Croydon, London, UK

^*^Both authors have contributed equally and should be recognised as co-first authors.

**Corresponding author:**

Harroop Bola

Imperial College School of Medicine, South Kensington, London SW7 5NH

+44 7873464967

Hb519@Ic.ac.uk

Acknowledgements

Source - UK Medical Education Database (“UKMED”) P134 extract generated on 16/06/2021. Approved for publication on 21/06/2024. We are grateful to UKMED for the use of these data. However, UKMED bears no responsibility for their analysis or interpretation. The data includes information derived from that collected by the Higher Education Statistics Agency Limited (“HESA”) and provided to the GMC (“HESA Data”). Source: HESA Student Record 2002/2003 and 2017/2018 Copyright Higher Education Statistics Agency Limited. The Higher Education Statistics Agency Limited makes no warranty as to the accuracy of the HESA Data, cannot accept responsibility for any inferences or conclusions derived by third parties from data or other information supplied by it.

**Supplementary Material - Index**

| **Supplementary Methods** | *pag. 3* |
| --- | --- |
| Background | *pag. 3* |
| Study Design | *pag. 4* |
| Statistical Analysis | *pag. 5* |
| Ethical Considerations | *pag. 6* |
| **Supplementary Results** | *pag. 7* |
| Cohort Characteristics | *pag. 7* |
| Variable Analysis | *pag. 7* |
| **Supplemental Appendixes** | *pag. 9* |
| Discussion and Limitations | *pag. 9* |
| **Supplemantary Figures and Tables** | *pag.12* |
| Figure S1 | *pag.12* |
| Table S1 | *pag. 13* |
| Table S2 | *pag. 14* |
| Table S3 | *pag. 15* |
| Table S4 | *pag. 17* |
| Table S5 | *pag. 18* |
| Table S6 | *pag. 19* |
| **References** | *pag. 20* |

**Background**

In the United Kingdom, newly qualified doctors will typically undertake a two-year long Foundation Training programme often comprising both medical and surgical rotations before specialising further. For those choosing to pursue a career in surgery, the next stage will typically be the Core Surgical Training (CST) programme, a further two-year long programme designed to equip doctors with formal training in a variety of surgical specialities enabling aspiring surgeons to learn surgical skills and build on existing surgical knowledge [1].

Securing a position as a core surgical trainee is competitive, with a growing number of applicants compared to the limited available posts [2]. In 2023, there were a total of 2539 applicants for 609 posts across the UK, resulting in a competition ratio of 4.17, a rise from 3.70 in 2022, and 2.93 in 2019 [3,4]. After successful CST completion, trainees will be eligible to apply for Higher Surgical Training (ST3) in their desired speciality. General surgery is one of the largest surgical specialities; however, the number of available ST3 posts has not adjusted to the increasing number of applicants in this speciality, with 607 applicants applying for 136 ST3 posts in 2021, leading to a competition ratio of 4.46, among the highest of all the specialities [5,6]. Despite the availability of data on competition ratios, there is a paucity of data on the demographic, socioeconomic and educational factors that are associated with applying and successfully obtaining an ST3 post.

Diversity in surgery has become a topic of interest in recent years, particularly with regard to sex disparity. Evidence has shown that although 59% of medical students are female, only 41% of core surgical trainees and 12% of consultant surgeons are female [7]. Notably, general surgery stands out as a specialty showing a positive trend in the number of female registrars and consultants in comparison to other surgical specialties. For example, in 2020, general surgery in the UK had 39.8% female registrars. In comparison, urology, vascular surgery, and neurosurgery had 31.6%, 25.0%, and 24.7% female registrars, respectively [8].

Despite existing analyses of general surgical trainees, data on the demographic, socioeconomic, and educational factors influencing applications and success in obtaining general surgery ST3 posts remain limited. This study aims to fill this gap by describing these factors for core surgical trainees applying for and receiving general surgery higher surgical training (ST3) posts.

**Methods**

**Design and study setting**

This is a retrospective study utilising data sourced from the UK Medical Education Database (UKMED). UKMED links data from existing routine data collections concerning medical students and trainee doctors across the United Kingdom. Permission to access and employ these data was granted by the UKMED Advisory Board, with the General Medical Council (GMC) serving as the data controller. The study protocol can be accessed in the supplementary materials online. The data in this study are derived from the UKMEDP134 extract, generated on 16/06/2021, and authorised for publication by UKMED on 01/12/2023. Our reporting adheres to the Strengthening the Reporting of Observational Studies in Epidemiology (STROBE) guidelines [9].

**Participants**

The population of this study comprised doctors who had completed the initial two years of foundation training as well as two years of core surgical training and were eligible to submit a first-time application for higher surgical training in the United Kingdom between 01/01/2014 and 31/12/2019.

**Study variables**

A description of the named variables is outlined in Table S1. Further details for the names of the variables can be found in the UKMED data dictionary [10].

The study examined various factors influencing medical trainees' applications for general surgery higher speciality training. Exposure variables included demographics factors such as age, sex and ethnicity; socioeconomic factors, and educational factors, such as medical school category (Russell group vs non-Russell group), Situational Judgement Test (SJT) scores at medical school and Membership of the Royal College of Surgeons (MRCS) success (Part A and B).

The primary outcomes were whether CSTs applied for a general surgery ST3 post and if they received an offer on their first attempt. Data were provided from 2014 to 2019, focusing on first time applications for general surgery training to ensure consistency. Data on simultaneous applications to other specialties were included and considered in the analysis.

To comply with the statistical disclosure controls of UKMED and the Higher Education Statistics Agency (HESA), all figures are rounded to the nearest multiple of five. Certain percentages, derived from a small sample size of fewer than 22.5 individuals and averages based on seven or fewer individuals were suppressed. Although the statistical analyses were conducted using the raw data, it is important to acknowledge that some figures and percentages might seem imprecise due to limitations imposed by HESA disclosure controls.

Several demographic factors were evaluated as binary variables, such as pre-medical school domicile (UK vs non-UK domicile). Additionally, Participation of Local Areas (POLAR) and Indices of Multiple Deprivation (IMD) were evaluated as ordinal variables. POLAR categorises areas based on the proportion of young people who participate in higher education, providing insight into educational access. IMD measures levels of deprivation across multiple domains such as income, employment and health, offering a comprehensive assessment of social disadvantage. These metrics helped assess the trainees' residential locations and the degree of social deprivation in those areas [10].

Pre-medical school education status was determined using HESA tariff points, which allocate points to prospective medical students based on their performance in post-16 exams, typically A-Levels. The calculation of the tariff score involves assigning numerical values to the type of qualification and grades achieved, with duplicate qualifications in the same subject excluded from the calculation [11].

Medical school educational status included the distinction between Russell Group and Non-Russell Group institutions. The Russell Group comprises a group of 24 self-selected UK universities known for their world-class and research-intensive programs, producing more than more than two-thirds of the world-leading research produced in UK universities [12]. Additionally, the Foundation Programme Application System (FPAS) Situational Judgement Test (SJT) score was considered as a continuous variable. The SJT assesses various domains such as teamwork, communication, and ethical judgment and is scored out of 50 points. Applicants were categorised into four groups based on their SJT scores. The number of SJT attempts was also recorded.

Pre-surgical training status was evaluated based on first-attempt scores relative to the pass mark for both MRCS Part A and Part B exams. The Annual Review of Competency Progression (ARCP) recruitment outcomes were assessed, with 'outcome 6' indicating that doctors have gained all required competencies and can progress to the next stage of training.

The analysis of ST3 specialty training applications included binary variables such as whether the trainee applied to general surgery higher specialty training, submitted applications to other specialties, and received an offer for a general surgery ST3 post.

**Statistical analysis**

The cohort characteristics were described in detail. Initial univariate logistic regression models were conducted to determine the odds ratios (OR) for the association between each exposure and the outcomes: application to and acceptance of a general surgery ST3 post. Subsequently, a directed acyclic graph (DAG) was employed to explore causal inferences and identify the relevant confounders for adjustment. Each factor was individually considered as the primary exposure variable in the model to determine the minimal adjustments required (see Figure S1 and Table S2). Logistic regression was then conducted for all exposures again to calculate the adjusted odds ratios, taking confounders into account as directed by the DAG. A 95% confidence interval was used to indicate significance. All analyses were performed using STATA 15.1 (Texas, US).

**Patient involvement**:

No patients were involved in this study

**Ethics and Data Access**:

Ethical approval was not deemed necessary for this study. The study utilised anonymous data from UKMED. The General Medical Council (GMC) acts as the data controller for UKMED and has a pre-existing, established research agreement and access arrangement for the use of this data. The Medical Schools Council (MSC) has previously confirmed that research projects using UKMED-held data are exempt from the requirement for ethics approval [13].

**Results**

**Cohort Characteristics**

The cohort characteristics are described in Tables S3 and S4. Of the 1960 core surgical trainees, the majority were born between 1980-1989 (1355/1960, 69.13%). Most were male (1240/1960, 63.27%), had resided in the UK prior to medical school (1580/1960, 80.61%), and more than half (1120/1960, 57.14%) were white.

Parental socioeconomic status (SES) was assessed as an indicator of overall socioeconomic status by categorising parents’ occupations for core surgical trainees. It was noted that one-third (32.91%) of core surgical trainees' parents were involved in higher managerial and professional occupations. Additionally, an investigation of the IMD demonstrated that again, one-third (31.63%) of all core surgical trainees were from the ‘least deprived area’ (Index 5). POLAR quintile analysis also revealed that 770 (39.29%) of core surgical trainees were categorised in the POLAR quintile 5 area, where quintile 5 represents an ‘extreme likelihood’ of younger people entering higher education. This finding suggests that trainees from more affluent backgrounds are likely overrepresented in surgical training programmes.

Most core surgical trainees (76.79%) did not hold a previous degree when applying for medical school, and the majority attended a Russell Group university (68.88%). The HESA tariff points were examined for core surgical trainees as a pre-medical school educational measure. More than half (53.32%) of core surgical trainees had 400-599 points, and 9.95% obtained between 600-799 points (Table S4). Additionally, MRCS Part A and Part B results were analysed for core surgical trainees. For MRCS Part A, 73.21% of trainees scored less than 20 points above the average score required to pass, and 3.57% scored between 40-59 points above the average score required to pass. Concerning MRCS Part B, 12.24% of trainees scored less than 20 points above the average score required to pass; however, 13.01% scored more than 60 points above the average score required to pass. These results indicate a wide range of performance levels among core surgical trainees on the MRCS exams.

**Factors associated with applying to General Surgery specialty training**

There were 1719 (87.70%) core trainees that successfully achieved ARCP outcome 6 and were, therefore, eligible to apply for higher surgical training (ST3) posts. Of the 1719 eligible applicants, 1196 (61.00%) were offered an ST3 post in any speciality. 706 (36.00%) applied for general surgery, the most popular of all specialities, and out of those who applied, 477 (67.56%) were offered an ST3 post.

Table S5 summarises our key findings. We identified an association between sex and applying for general surgery with 320/720 female CSTs that applied for general surgery, compared to 380/1240 male CSTs, favouring women (OR=1.82, 95% CI=1.51-2.20). Age and ethnicity, however, were not associated with increased odds of applying to general surgery specialty training.

Additionally, we found an association between being from IMD quintile 2 (the second most deprived area) and applying to general surgery, in comparison to those from IMD quintile 1 (the most deprived area) (OR=1.98, 95% CI=1.12-3.46). A third association was found, between domicile and applying for general surgery, favouring core surgical trainees who were domiciled outside of the UK prior to medical school (OR=0.60, 95% CI=0.44-0.82). None of the other variables of interest were associated with increased likelihood of applying for general surgery training.

**Factors associated with being offered a general surgery ST3 post**:

Of the 706 first time general surgery applicants, 477 (67.56%) were offered an ST3 post. Age was not associated with increased odds of being offered a post. There was, however, an association between sex and being offered a post, favouring women – 239/323 (73.99%) of female applicants were offered a post, compared to 238/383 (62.14%) of male applicants (OR=1.73, 95% CI=1.25-2.39). Ethnicity was also found to have a significant association with increased odds of being offered a post, favouring applicants identifying as white – 277/391 (70.84%) of white applicants were offered a post, compared to 172/271 (63.47%) of ethnic minority applicants (OR=0.72, 95% CI=0.51-0.99).

In terms of educational factors, core surgical trainees who attended Russell Group universities for medical school were nearly twice as likely to secure an offer for a higher surgical training post in general surgery compared to those from non-Russell Group medical schools. Specifically, 342/464 trainees (73.71%) from Russell Group universities obtained a higher general surgery post, compared to 91/152 trainees (59.87%) from non-Russell Group universities. (OR = 2.22, 95% CI = 1.41-3.48).

Following this, those that achieved 35-39 (140/186, 75.27%) and 40-44 (135/196, 68.88%) on their SJT were approximately three times more likely to be offered an ST3 post in comparison to those that achieved <35 out of a maximum score of 50. (OR=3.67, 95% CI=1.98-12.50), (OR=3.41, 95% CI=1.02-11.47).

**Multiple applications**

Of the 706 first-time general surgery applicants, 65 (9.21%) simultaneously applied to more than one specialty in the same recruitment round. However, applying to multiple specialties did not increase the odds of receiving an offer, as none of these applicants were offered a post, as shown in Table S6.

**Discussion**

The study aimed at investigating the characteristics of applicants who apply to and are successful in obtaining higher surgical training (ST3) posts in General Surgery in the UK. We found that being female or residing outside of the UK both had a positive association with the odds of applying for general surgery training. With regards to successful applicants, we identified positive associations favouring being female, white, attending a Russell Group university and achieving a high SJT score.

This study suggests that females were more likely to apply and be offered a general surgery post in comparison to their male counterparts while surgery still tends to be male dominated, there is an increasing body of literature surrounding sex disparity, with important efforts to increase diversity. A previous observational study using NHS data between 2011-2020 demonstrated a positive trend in female representation for both registrars and consultants in general surgery, with general surgery estimated to achieve parity between sexes by 2028, compared to Neurosurgery, Trauma & Orthopaedics and Cardiothoracic Surgery which are estimated to take 45-60 years [8]. Key initiatives to increase the number of females in general surgery include: The Women in Surgery (WinS) forum to support females in surgery [14], initiatives to improve work-life balance e.g. allowing trainees the option to work less than full time and finally mentorship. One systematic review has provided evidence that mentorship has been recognised as a key factor in inspiring junior females towards pursuing surgery [15]. Further qualitative research could be helpful in determining why being a female trainee is associated with greater odds of applying to and receiving offers for general surgery as identified in this study.

The encouraging results regarding sex diversity are a cause for cautious optimism. However, general surgery recruitment continues to face challenges in other areas, particularly with respect to ethnic diversity. Our study found an association between ethnicity and being offered an ST3 post, favouring white candidates. This disheartening statistic is unfortunately supported by further evidence in the field. A studying the US found that more than 50% of general surgery residents reported experiencing some form of racial mistreatment, suggesting pervasive racial discrimination in the profession [16].

Furthermore, while NHS workforce data reveal that 50% of all UK registered doctors are white, with the other 50% identifying as Ethnic Minority (EM) [17]. EM doctors across specialties continue to have lower pass rates in specialty exams, a higher chance of obtaining an unsatisfactory ARCP outcome and remain underrepresented in surgical leadership [18,19]. While being domiciled outside of the UK prior to medical school was positively associated with applying for ST3, it did not affect the odds of obtaining an offer. To our knowledge, no existing literature explores why international medical students choose specific specialties. This suggests that the relationship between country of origin, ethnicity, and the likelihood of entering surgical training is complex. There is evidence that being an ethnic minority makes one subject to discrimination and less likely to obtain a general surgery offer; however, this is not the case for international students.

Educational factors were also explored in this study. It was found that attending a Russell Group university for medical school was associated with higher odds of obtaining an offer for a General Surgery ST3 post. Evidence indicates that success in the MRCS, a prerequisite for obtaining an ST3 post, is linked to the medical school attended. One study found that attending a Russell Group university predicted MRCS success (p<0.05) [20]. While our study showed that an SJT score between 35 and 44 was associated with better odds of obtaining a general surgery offer, no prior studies have examined whether SJT scores impact success in obtaining an ST3 higher surgical training post. Existing literature only identifies the SJT as a predictor of success in the foundation programme, and little research has been conducted on the SJT as a predictor of success in surgical training. In fact, one study indicated that the SJT score was not a statistically significant independent predictor of MRCS success [20]. It is worth noting however, that as of 2024, the UK Foundation Programme allocation process has entirely removed the SJT in favour of a "preference informed allocation" system [21]. Under this system, students are randomly assigned a rank generated by a computer. This change suggests a potential shift in focus towards alternative metrics or methods for evaluating candidates for the Foundation Programme and potentially for surgical training in the future.

Strengths of this study include its large sample size and detail of available information, utilising longitudinal data of 1960 trainees. We were able to study long-term trends for different factors, comparing those that applied for a training post and those that were offered a post. As the UKMED database was utilised, comprising data collected by the GMC as part of their statutory duty to regulate medical education in the UK, this study did not have to rely on the voluntary completion of surveys. Quantitative data were utilised and processed via logistic regression analysis to establish relevant associations, while accounting for confounders.

The most important limitation of this study is that our population only comprised applications submitted by doctors who obtained their degree in the UK and had completed CST. Doctors who qualified abroad (International Medical Graduates, or IMGs) or applied to HST via alternative routes, e.g., Certificate of Readiness to Enter Specialty Training, were not included. It is important to note that HEE has started reporting statistics on country of qualification, showing that for the 2023 year 100 general surgery ST3 offers were made for 160 UK-based applicants, 13 offers for 27 EU-based applicants and 79 offers for 328 applicants from the rest of the world [22]. Future studies could therefore address dividing IMGs versus UKGs and investigate any disparities that may exist between the cohorts.

Additionally, there were varying degrees of missing data for each factor assessed, as recorded in the results, particularly SES and SJT scores. It is recognised that ethnicity was also only explored in a binary fashion (White vs Ethnic Minority) rather than with a more granular approach which would be beneficial in future research. Furthermore, this data does not account for attrition rates of trainees and assumes all trainees who were offered a post accepted the post.

Another important limitation is the fact that we excluded repeat applicants from our analysis; it is known that a significant bottleneck exists at ST3 stage, with many applicants only obtaining an offer on their second or third attempt. Moreover, due to governance processes and internal peer review within UKMED, there was an extended delay between the completion of the study and permission for publication. As such, all data analysed predates the COVID-19 pandemic, which has since had a significant impact on surgical training in the UK, including both the application and recruitment processes. Future studies are required to add to this existing evidence and provide a more up-to-date reflection of the current state of surgical training.

**Conclusion**

Overall, we found an association between sex and applying for general surgery ST3, favouring female trainees. Additionally, there was an association between sex and receiving an offer for general surgery ST3 training, also favouring female trainees. We also observed an association between ethnicity and receiving an offer, favouring white applicants, in contrast to domicile status, which did not reveal increased odds of success. This mixed picture highlights some of the successes of equality, diversity and inclusion initiatives while also providing evidence of ethnic disparity within UK general surgical training. More work is needed to ensure the formation of a diverse surgical workforce.

**Supplementary Figures and Tables**


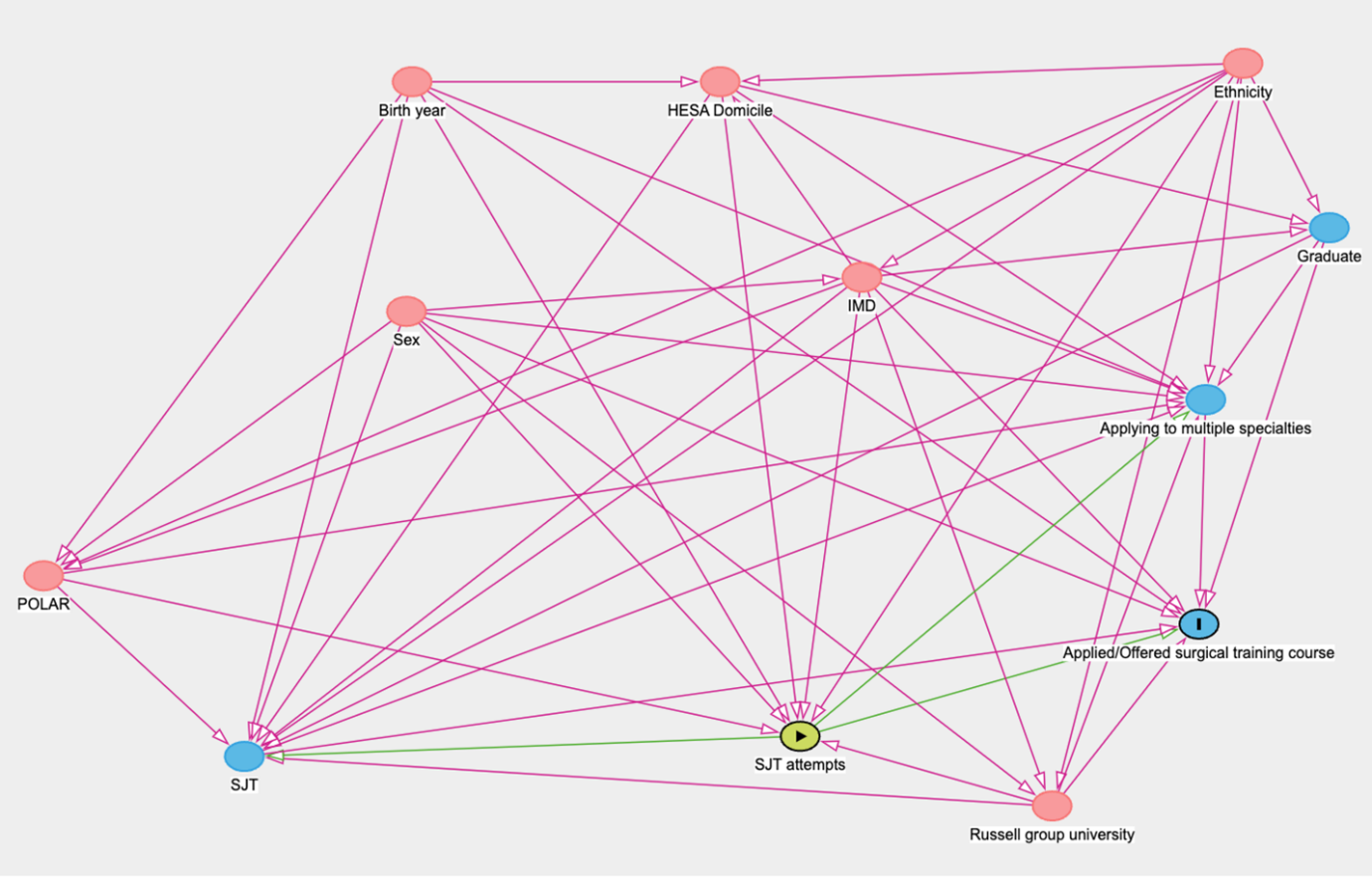


**Figure S1** - Directed acyclic graph (DAG) to determine the confounding factors

**Table S1 -** Description of included variables

| **Variable group** | **Factor** | **Level** | **Description** | **Missing data** |
| --- | --- | --- | --- | --- |
| Demographics | Year of birth | Ordinal | Min = 1960s  Max = 1990s | N=0 |
|  | Sex | Binary | Male vs female | N=0 |
|  | Ethnicity | Binary | Black and minority ethnic (BAME) vs white | N=105, 5.36% |
|  | Pre-medical school domicile | Binary | UK vs non-UK domicile | N=180, 9.18% |
|  | Parental Socioeconomic Status (SES) | Discontinuous | Occupation titles | N=190, 9.69% |
|  | Participation of local areas (POLAR) | Continuous | Min = 1  Max = 5 | N=390, 19.90% |
|  | Indices of multiple deprivation (IMD) | Continuous | Min = 1  Max = 5 | N=390, 19.90% |
| Pre-medical school educational status | Higher Education Statistics Agency (HESA) tariff points | Continuous | Min = 0  Max = 999 | N=185, 9.43% |
|  | University degree attained prior to medical school | Binary | Degree attained vs no degree | N=180, 9.18% |
| Medical school educational status | FPAS Situational Judgement Test score | Continuous | Min = <35  Max = >45 | N=725, 36.99 % |
|  | Medical School Category | Binary | Russell Group vs Non-Russell Group | N=180, 9.18% |
| Pre-surgical training status | First attempt score on the MRCS Part A exam (relative to average pass mark) | Continuous | Min = <20  Max = 40-59 | N=0 |
|  | First attempt score on the MRCS Part B exam (relative to average pass mark) | Continuous | Min = <20  Max = 60+ | N=20, 1.02% |
| Speciality training application (ST3) | Eligible to apply to speciality training | Binary | Achieving outcome 6 on ARCP vs not achieving outcome 6 on ARCP | N=90, 4.59% |
|  | Applied to at least one of the seven uncoupled surgical training programmes | Binary | Applied vs not applied | N=0 |
|  | Offered at least one of the seven uncoupled surgical training programmes | Binary | Offered vs not offered | N=0 |
|  | Applied to T&O higher surgical training | Binary | Applied vs not applied | N=0 |
|  | Offered a T&O higher surgical training post | Binary | Offered vs not offered | N=0 |

**Table S2** - Exposures and the minimal adjustments required

| **Exposure** | **Minimal adjustments required according to DAG** |
| --- | --- |
| Birth year | No adjustments needed |
| Sex | No adjustments needed |
| Ethnicity | No adjustments needed |
| IMD (quintile) | Ethnicity, sex |
| HESA Domicile | Birth year, ethnicity, IMD |
| Graduate on entry | Ethnicity, HESA, IMD |
| POLAR (quintile) | Birth year, ethnicity, sex |
| Russell group medical school | Ethnicity, IMD, sex |
| SJT score | Birth year, graduate, Russell group, IMD, SJT attempts, sex, ethnicity, HESA, POLAR |
| SJT number of attempts | Birth year, ethnicity, HESA, POLAR, Russell group, IMD, sex |
| Reapplication | Birth year, graduate, IMD, Russell group, SJT score, SJT attempt, sex |

**Table S3** - Demographics of the Core Surgical Trainee cohort (n=1960)

|  | **Total n=1960** | |
| --- | --- | --- |
|  | n | % |
| **Birth decade** |  |  |
| 1960-1969 | 0 | N/A^†^ |
| 1970-1979 | 15 | N/A^†^ |
| 1980-1989 | 1,355 | 69.13 |
| 1990 onwards | 585 | 29.85 |
|  |  |  |
| **Sex** |  |  |
| Male | 1,240 | 63.27 |
| Female | 720 | 36.73 |
|  |  |  |
| **Ethnicity** |  |  |
| White | 1,120 | 57.14 |
| BME | 735 | 37.50 |
| Missing | 105 | 5.36 |
|  |  |  |
| **Domicile** |  |  |
| UK | 1,580 | 80.61 |
| Non-UK | 205 | 10.46 |
| Missing | 180 | 9.18 |
|  |  |  |
| **Parent's Socioeconomic Status (SES)** |  |  |
| Higher managerial & professional occupations | 645 | 32.91 |
| Intermediate occupations | 155 | 7.91 |
| Lower managerial & professional occupations | 320 | 16.33 |
| Lower supervisory & technical occupations | 30 | 1.53 |
| Never worked & long-term unemployed | 0 | N/A^†^ |
| Routine occupations | 25 | 1.28 |
| Semi-routine occupations | 100 | 5.10 |
| Small employers & own account workers | 70 | 3.57 |
| Other | 425 | 21.68 |
| Missing | 180 | 9.18 |
|  |  |  |
| **Participation of Local Areas (POLAR)** |  |  |
| 1 | 55 | 2.81 |
| 2 | 130 | 6.63 |
| 3 | 245 | 12.50 |
| 4 | 370 | 18.88 |
| 5 | 770 | 39.29 |
| Missing | 390 | 19.90 |
|  |  |  |
| **Index of Multiple Deprivation (IMD)** |  |  |
| 1 | 105 | 5.36 |
| 2 | 160 | 8.16 |
| 3 | 290 | 14.80 |
| 4 | 395 | 20.15 |
| 5 | 620 | 31.63 |
| Missing | 390 | 19.90 |
| ^†^Denotes percentages based on fewer than 22.5 individuals, which are suppressed as per HESA statistical disclosure controls. | | |

**Table S4** - Educational factors analysed for the Core Surgical Trainee cohort (n=1960)

|  | **Total n=1960** | |
| --- | --- | --- |
| **HESA Tariff Points** | n | % |
| 0-199 | 385 | 19.64 |
| 200-399 | 150 | 7.65 |
| 400-599 | 1,045 | 53.32 |
| 600-799 | 195 | 9.95 |
| 800-999 | 5 | N/A† |
| Missing | 180 | 9.18 |
|  |  |  |
| **Graduate on entry** |  |  |
| Graduate on entry | 270 | 13.78 |
| Not graduate on entry | 1,505 | 76.79 |
| Missing | 185 | 9.44 |
|  |  |  |
| **Russell Group Medical School** |  |  |
| Non-Russell Group | 430 | 21.94 |
| Russell Group | 1,350 | 68.88 |
| Missing | 180 | 9.18 |
|  |  |  |
| **Number of SJT attempts** |  |  |
| 1 | 1525 | 77.81 |
| 2 | 10 | N/A^†^ |
| Missing | 420 | 21.43 |
|  |  |  |
| **SJT score** |  |  |
| <35 | 85 | 4.34 |
| 35-39 | 555 | 28.32 |
| 40-44 | 545 | 27.81 |
| >=45 | 45 | 2.30 |
| Missing | 725 | 36.99 |
|  |  |  |
| **MRCS part A*** |  |  |
| <20 | 1,435 | 73.21 |
| 20-39 | 460 | 23.47 |
| 40-59 | 70 | 3.57 |
|  |  |  |
| **MRCS part B*** |  |  |
| <20 | 240 | 12.24 |
| 20-39 | 720 | 36.73 |
| 40-59 | 730 | 37.24 |
| 60+ | 255 | 13.01 |
| Missing | 20 | N/A^†^ |
| ^*^For MRCS, the categories correlate to number of points scored above the average score required to pass.  ^†^Denotes percentages based on fewer than 22.5 individuals, which are suppressed as per HESA statistical disclosure controls. | | |

**Table S5 -** Odds ratios and 95% confidence intervals (CIs) for factors associated with applying to a higher surgical training post in General Surgery (n=705)

| **Variable** | **Number of applicants** | **OR^‡^** | **95% CI** |
| --- | --- | --- | --- |
| **Birth decade** |  |  |  |
| 1960s | 0 | 1.00 | Ref |
| 1970s | 10 | 0.89 | (0.05-16.67) |
| 1980s | 480 | 0.55 | (0.03-8.89) |
| 1990s | 215 | 0.57 | (0.04-9.18) |
|  |  |  |  |
| **Sex** |  |  |  |
| Male | 385 | 1.00 | Ref. |
| Female | 325 | 1.82**^*^** | (1.51-2.20) |
|  |  |  |  |
| **Ethnicity** |  |  |  |
| White | 385 | 1.00 | Ref. |
| BME | 270 | 1.08 | (0.89-1.32) |
|  |  |  |  |
| **IMD** |  |  |  |
| 1 | 30 | 1.00 | Ref. |
| 2 | 70 | 1.98**^*^** | (1.12-3.46) |
| 3 | 90 | 1.09 | (0.65-1.85) |
| 4 | 125 | 1.19 | (0.71-1.98) |
| 5 | 200 | 1.15 | (0.71-1.88) |
|  |  |  |  |
| **Domicile** |  |  |  |
| Non-UK | 90 | 1.00 | Ref. |
| UK | 525 | 0.60**^*^** | (0.44-0.82) |
|  |  |  |  |
| **Graduate on entry to medical school** |  |  |  |
| Graduate | 100 | 1.00 | Ref. |
| Not a graduate | 515 | 0.92 | (0.60-1.43) |
|  |  |  |  |
| **POLAR quintile** |  |  |  |
| 1 | 15 | 1.00 | Ref. |
| 2 | 40 | 1.00 | (0.48-2.04) |
| 3 | 100 | 1.39 | (0.72-2.70) |
| 4 | 120 | 0.99 | (0.52-1.88) |
| 5 | 240 | 0.96 | (0.51-1.79) |
|  |  |  |  |
| **Russell Group Medical School** |  |  |  |
| Russell Group | 465 | 1.00 | Ref. |
| Non-Russell Group | 150 | 1.03 | (0.79-1.33) |
|  |  |  |  |
| **SJT score** |  |  |  |
| <35 | 20 | 1.00 | Ref. |
| 35-39 | 185 | 1.17 | (0.64-2.16) |
| 40-44 | 195 | 1.32 | (0.72-2.44) |
| >=45 | 20 | 2.30 | (0.98-5.43) |
|  |  |  |  |
| **SJT Attempts** |  |  |  |
| 1 | 520 | 1.00 | Ref. |
| 2 | 0 | 0.35 | (0.04-2.95) |
|  |  |  |  |
| **Multiple Applications** |  |  |  |
| Applied to 1 specialty | 640 | 8.19^§^ | (3.47-11.70) |
| Applied to >1 specialty | 65 | 1.00 | Ref. |
| **^‡^**Denotes adjusted odds ratio(s) as defined by the DAG graph (Figure S1)  ^*^Denotes statistical significance i.e. *p<0*.*05*  ^§^Denotes cases where the presence of collinearity rendered precise estimation unattainable. | | | |

**Table S6** – Odds ratios and 95% confidence intervals (CIs) for factors associated with being offered a higher surgical training post in General Surgery (n= 475)

| **Variable** | **Number offered** | **OR^‡^** | **95% CI** |
| --- | --- | --- | --- |
| **Age decade** |  |  |  |
| 1960s | 0 | N/A | N/A |
| 1970s | 5 | 1.00 | Ref. |
| 1980s | 330 | 2.16 | (0.53-8.77) |
| 1990s | 140 | 2.00 | (0.49-8.23) |
|  |  |  |  |
| **Sex** |  |  |  |
| Male | 240 | 1.00 | Ref. |
| Female | 240 | 1.73**^*^** | (1.25-2.39) |
|  |  |  |  |
| **Ethnicity** |  |  |  |
| White | 275 | 1.00 | Ref. |
| BME | 170 | 0.72^*^ | (0.51-0.99) |
|  |  |  |  |
| **IMD** |  |  |  |
| 1 | 20 | 1.00 | Ref. |
| 2 | 40 | 0.83 | (0.32-2.16) |
| 3 | 70 | 1.33 | (0.51-3.45) |
| 4 | 95 | 1.36 | (0.55-3.41) |
| 5 | 140 | 1.20 | (0.50-2.89) |
|  |  |  |  |
| **Domicile** |  |  |  |
| UK | 375 | 1.00 | Ref. |
| Non-UK | 60 | 1.46 | (0.89-2.42) |
|  |  |  |  |
| **Graduate on entry to medical school** |  |  |  |
| Graduate | 65 | 1.00 | Ref. |
| Not a graduate | 370 | 0.83 | (0.38-1.83) |
|  |  |  |  |
| **POLAR quintile** |  |  |  |
| 1 | 15 | 1.00 | Ref. |
| 2 | 25 | 0.66 | (0.36-3.85) |
| 3 | 65 | 0.61 | (0.33-2.93) |
| 4 | 85 | 0.88 | (0.32-3.56) |
| 5 | 180 | N/A | N/A |
|  |  |  |  |
| **Russell Group Medical School** |  |  |  |
| Non-Russell Group | 95 | 1.00 | Ref. |
| Russell Group | 340 | 2.22**^*^** | (1.41-3.48) |
|  |  |  |  |
| **SJT score** |  |  |  |
| <35 | 25 | 1.00 | Ref. |
| 35-39 | 140 | 3.67**^*^** | (1.98-12.50) |
| 40-44 | 135 | 3.41**^*^** | (1.02-11.47) |
| >=45 | 10 | 1.77 | (0.58-13.40) |
|  |  |  |  |
| **SJT Attempts** |  |  |  |
| 1 | N/A | 1.00 | Ref. |
| 2 | N/A | N/A | N/A |
|  |  |  |  |
| **Multiple Applications** |  |  |  |
| Applied to 1 specialty | 40 | 1.00 | Ref. |
| Applied to >1 specialty | 0 | 0.33 | (0.14-0.79) |
| **^‡^**Denotes adjusted odds ratio(s) as defined by the DAG graph (Figure 1)  ^*^Denotes statistical significance i.e. *p<0*.*05* | | | |

**References**

[1] Mellor K, Robinson D, James O, Powell A, Bowman C, Hopkins L, et al. Improving surgical training: core programme performance related to rotation theme, design and trainee protocol engagement. Bulletin 2021;103:061–8. https://doi.org/10.1308/rcsbull.TB2021.16.

[2] Faderani R, Abdi Z, Hastings A, Reed T, Zargaran D, Mosahebi A. Core surgical training: The influence of time and date on interview outcome. The Surgeon 2023;21:208–16. https://doi.org/10.1016/j.surge.2022.10.003.

[3] Core Surgical Training CT1 Competition Ratios n.d. https://www.specialty-applications.co.uk/competition-ratios/core-surgical-training-competition-ratios/#google_vignette.

[4] NHS England. Competition ratios for 2021 n.d. https://medical.hee.nhs.uk/medical-training-recruitment/medical-specialty-training/compehttps://medical.hee.nhs.uk/medical-training-recruitment/medical-specialty-training/competition-ratios/2021-competition-ratiostition-ratios/2021-competition-ratios.

[5] Torjesen I. Specialty training: record number of applicants and posts filled in England, say officials. BMJ 2022:o1649. https://doi.org/10.1136/bmj.o1649.

[6] NHS Education England Competition Ratios Nationally Advertised Vacancies (2021) n.d. https://specialtytraining.hee.nhs.uk/Portals/1/2021%20Competition%20Ratios_1.pdf.

[7] Moberly T. Number of women entering medical school rises after decade of decline 2018:k254.

[8] Newman TH, Parry MG, Zakeri R, Pegna V, Nagle A, Bhatti F, et al. Gender diversity in UK surgical specialties: a national observational study. BMJ Open 2022;12:e055516. https://doi.org/10.1136/bmjopen-2021-055516.

[9] Elm EV, Altman DG, Egger M, Pocock SJ, Gøtzsche PC, Vandenbroucke JP. Strengthening the reporting of observational studies in epidemiology (STROBE) statement: guidelines for reporting observational studies. BMJ 2007;335:806–8. https://doi.org/10.1136/bmj.39335.541782.AD.

[10] Smith D, Bloxham A, Woodward C, Gurusamy G. UKMED Data Dictionary 2024.

[11] HESA Student Record 2007/08. 2008. https://www.hesa.ac.uk/collection/c07051/tariff (accessed March 18, 2024).

[12] Russell Group | Our universities. 2024. https://russellgroup.ac.uk/about/our-universities/ (accessed February 18, 2024).

[13] Dowell J, Cleland J, Fitzpatrick S, McManus C, Nicholson S, Oppé T, et al. The UK medical education database (UKMED) what is it? Why and how might you use it? BMC Med Educ 2018;18:6. https://doi.org/10.1186/s12909-017-1115-9.

[14] Skinner H, Bhatti F. Women in surgery. Bulletin 2019;101:12–4. https://doi.org/10.1308/rcsbull.TB2019.12.

[15] Haque S. 634 Future Women in Surgery and the Role of Mentorship. British Journal of Surgery 2022;109:znac269.229. https://doi.org/10.1093/bjs/znac269.229.

[16] El Boghdady M, Ewalds-Kvist BM. Racial discrimination in surgery: A systematic review. Updates Surg 2023;75:795–806. https://doi.org/10.1007/s13304-023-01491-x.

[17] NHS Digital. NHS workforce 2023. https://www.ethnicity-facts-figures.service.gov.uk/workforce-and-business/workforce-diversity/nhs-workforce/latest/.

[18] General Medical Council. Tackling disadvantage in medical education. 2023.

[19] Joseph J, Joseph A, Jayanthi N, Pereira B, Gahir J. BAME underrepresentation in surgery leadership in the UK and Ireland in 2020: an uncomfortable truth. Bulletin 2020;102:232–3. https://doi.org/10.1308/rcsbull.2020.166.

[20] Ellis R, Scrimgeour DSG, Brennan PA, Lee AJ, Cleland J. Does performance at medical school predict success at the Intercollegiate Membership of the Royal College of Surgeons (MRCS) examination? A retrospective cohort study. BMJ Open 2021;11:e046615. https://doi.org/10.1136/bmjopen-2020-046615.

[21] UKFP 2024 Key Changes. UK Foundation Programme 2023. https://foundationprogramme.nhs.uk/programmes/2-year-foundation-programme/eligibility-information/ukfp-2024-key-changes/ (accessed March 23, 2024).

[22] NHS England. Country of qualification 2023 recruitment data n.d.
